# Supplementary material for: Improved prediction of antibody and their complexes with clustered generative modelling ensembles
Source: Bioinform Adv. 2025 Jul 3;5(1):vbaf161. doi: 10.1093/bioadv/vbaf161 (PMC12279294; doi:10.1093/bioadv/vbaf161)
Supplement: vbaf161_Supplementary_Data [file vbaf161_supplementary_data.pdf]

Supplementary Material

Improved structural modelling of antibodies and their complexes with clustered diffusion ensembles

X. Xu[1†], M. Giulini[1†], A.M.J.J. Bonvin [1]*

[1]Computational Structural Biology Group, Department of Chemistry, Bijvoet Centre, Faculty of Science, Utrecht University

† These authors contributed equally to this work.

*To whom correspondence should be addressed: a.m.j.j.bonvin@uu.nl

Supplementary Figure 1. Best ranked loops in terms of loop PLDDT 2

Supplementary Figure 2. Scatter plot of the highest H3 pLDDT versus the corresponding H3-RMSD value. 3

Supplementary Figure 3. Heavy chain CDR loop RMSD distribution of 1000 sampling on LOW-80-W dataset for BioEmu and AlphaFlow 3

Supplementary Table 1. CDR loop sequences of the 54 antibodies present in the data set 4

Supplementary information 1. An example HADDOCK configuration file for docking 5

Supplementary Figure 1. Best ranked loops in terms of loop PLDDT

We report here the RMSD values of the loop with the highest H3 loop pLDDT. Although the latter is a local measure tailored to predict the quality of the loop, it seems to perform slightly worse than the global Alphafold2 ranking score.

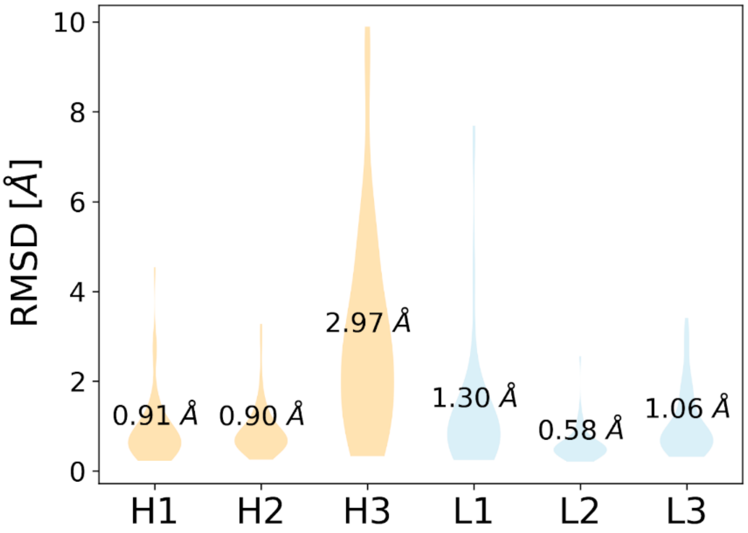

SI Figure 1. Violin plot of loop accuracy (measured by the loop RMSD from the reference crystal structure after superimposition on the frame-work region) over the six antibody hypervariable loops for the loops with the highest H3 loop pLDDT.

Supplementary Figure 2. Scatter plot of the highest H3 pLDDT versus the corresponding H3-RMSD value.

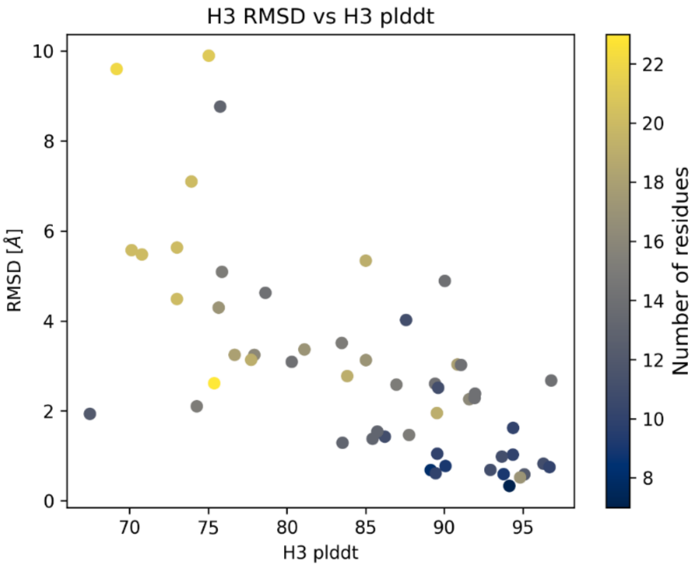

SI Figure 2. Scatter plot of the highest H3 pLDDT versus H3-RMSD A clear anti-correlation exists between the two (Pearson R=-0.67), especially in the right region of the figure, where high values of H3-pLDDT almost always correspond to good accuracy. The scenario is different for lower pLDDTs, where the H3-RMSD can be either low or high at the same pLDDT value. The circles are color-coded according to the length of the H3 loop

Supplementary Figure 3. Heavy chain CDR loop RMSD distribution of 1000 sampling on LOW-80-W dataset for BioEmu and AlphaFlow

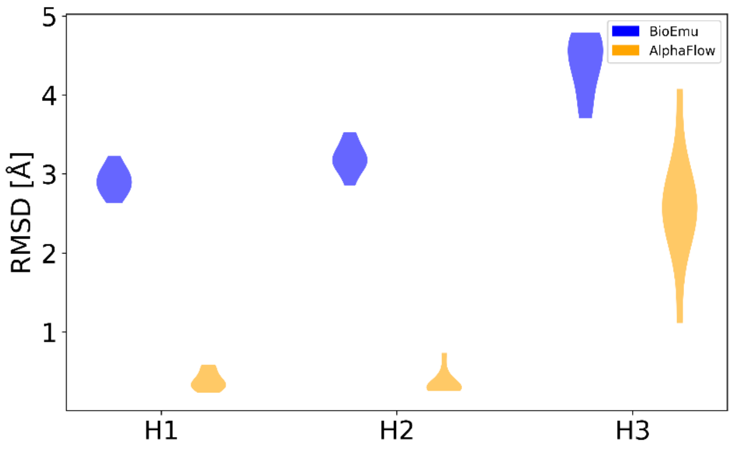

SI Figure 3. Violin plot comparing the best heavy chain loop RMSD from 1000 sampling from BioEmu and AlphaFlow on the LOW-80-W dataset

Supplementary Table 1. CDR loop sequences of the 54 antibodies present in the data set

PDB	H1	H2	H3	L1	L2	L3
7bbj	GYTFTTYW	IYPGLSDT	ARLLDYAMDY	QDIRSY	YTS	QQGETLPWT
7bnv	GYSFTSYW	IYPGDSDT	ARHPSPYYYGSGSYSGGFDY	QSVSSSY	GAS	QQYDNWPLMHT
7daa	GFSLSDYA	IYASGST	ARYYAGSDI	QSISAY	DAS	QTYYAIITYGAA
7dk2	GFTFSSYW	IKQDASEK	ARDLGILWFGDYP	QGISNS	AAS	QQFYSTPRT
7e72	GYSFTSYW	IHPSDSET	ARGLYGNS	QDIGIS	ATS	LQYASSPYT
7f7e	GFTFSSYA	IVGSGGST	AKSLIYGHYDILTGAYYFDY	QGIGNW	AAS	QQANSFPP
7kez	GFNIKDTY	IYPTNGYT	ARGGAVAGTGVYYFDY	QDIPRSISGY	WGS	QQHYTTPPT
7kf0	GFNIKDTY	IYPTNGYT	ARGGSFYYYYMDV	QDIPRSISGY	WGS	QQHYTTPPT
7kf1	GFNIKDTY	IYPTNGYT	AKLGIGYYYYGMDV	QDIPRSISGY	WGS	QQHYTTPPT
7kql	GGSISSRSYY	IYYSGFT	ATGGPYGDYAHWFEP	QSVSSSY	GAS	QQYGSSPIT
7l7r	GFTFSSYV	IYRGGST	VKDPKAWLEPEW	QSISKY	AAS	QQSYSNPRT
7lr3	EFTFSDYG	ISSGSNSI	SREAYFAMDY	QDIHKY	YTS	LQYDNLYT
7lr4	DYSLSDYN	INPNHGTT	ASPIHYGNHVPFDY	QDISNY	FTS	QQGITLPWT
7mdj	GYTFTSDW	IIPSYGRA	ARERGDGYFDY	QSIGTD	YAS	QQSNRWPFT
7mrz	GGSISSSSYY	ISYSGST	ARDSLRYGMDV	QSVLYSSNNKNY	WAS	QQYALAPPRT
7msq	GGSISSYH	IYYSGNT	VREMRRGYSGYDYWDLYAFDI	QGISSY	AAS	QQLNSYPHT
7mzf	EFIVSRNY	IYSGGTT	ARDRGDYLFDY	QSISSW	KAS	QQYNSYFPT
7mzg	GLTVSSNY	FYPGGST	ARDAVYYGMDV	QSISSY	AAS	QESYSTPGLFT
7mzh	GYTFTGYY	INPNSGGT	ARSYYDY	SSNIGHNA	YDD	AAWDDILNGPV
7mzi	GFTFSRFA	ISGSGGST	AKVGWGAFDI	YSNIGSNP	AND	STWDDSLPGPL
7mzj	GFTFSYAW	IKRKSDGGTT	TTDLCRSTSCEHDAFDI	QSIRSY	AAS	QQSYTTPAIT
7mzk	GYTFTSYY	INPSGGGT	AKDRVTIFWGNGMDV	QSVLYSSNNKNY	WAS	HQYSTTPLT
7n4i	GYSFISYW	IYPGDSDT	ARLLYYSDSSPLDS	QSISTY	AAS	QQSHSTPRT
7n4j	GDSISSSDYS	IYYIKNT	ARERPPFDVVVVPAARPYNWFDP	SSNIGAGYD	GNN	QSYDSSLSGSKV
7np1	GITVSSNY	IYSGGST	ARGEGGSIVGVTSDY	QSISRY	AAS	QQSYSTLPYT
7nx3	GYAFSSYW	IYPGDGDT	ARSRGYFYGSTYDS	ESVDNYGISF	AAS	QQSKEVPWT
7phu	GYTFTDYY	INPNSGGT	ARDLWFGESPPYYGVDV	NIGSYS	YDS	QVWDTNTDHVV
7phw	GFDFSSYA	IRNDGSFT	TKSADDGGHYSDFSGEIDA	TYNY	YND	GNSDSRNVA
7pi7	GFNIKDTY	IDPANGNT	ARDVLYFDV	ESVDSYGNSF	RAS	QQSNEDRT
7pqy	ASGFTVSSNYMS	SVIYSGGSTYY	YCARDHVRPGMNIWG	CQASQDISNY	LLIYDAS	ATYYCQQYDNLPV
7pr0	GFTFSSYS	ISSSSSTI	ASPGGITAAGTSVLFGYYGMDV	QSLLHSNGYNY	LGS	MQALQTPITWT
7ps0	DGSISSSDYY	IYYTGST	ARLVVPSPKGSWFDP	SIDVGNYNL	EGS	CSYVGSSTYV
7ps1	GLTVRSNY	IYSGGST	ARDLVVYGMDV	QSVSSSS	GTS	QQYGSSPL
7ps2	GFTFSNYG	ISYEESNR	AKDQGPATVMVTAIRGAMDV	QSVLYSSNNKNY	WAS	QQYFGSPSIT
7ps4	GYSFTNYW	IYPGDSGT	ARSRVGATGGYYDYYMDV	SSNLGGNT	SNN	AAWDDSLNGPV
7ps6	GGSITSSNHY	MYYSGST	ARQIGPKRPSQVADWFDP	QGISSY	AAS	QQLNSYPLT
7q0g	GGTFSSSV	IIPLFGSA	AKVSQWALILF	QSVSSSY	GAS	QQYGTSPSWT
7q0i	GFTFSSYG	IWYDGSNN	ARSYCSGGFCFGYYYGLDV	NIGTKS	YNS	QVWDSGSDHYV
7qnw	GDSISSSRYY	FYYSGIT	ARPRPPDYYDNSGALLFDI	QSISAW	KAS	QQYISSSPWT
7qny	GFTFDDYA	VSWNSGTI	AREVGGTFGVLISREGGLDY	TIGSKS	DDS	QVWDSSSDRVV
7qu1	GYAFGSHW	IYPGDGDT	ARDDYGTRYYFDY	QDINNY	YTS	QQGKTLPLT
7qu2	GFTFSNYQ	ITVKSDNYGA	SRSGIYDGYYAYAMDY	QIVGTS	WAS	QQYATYPLT
7rfb	GGTVNT	IFPLLGVP	AKDGVGWSGHGPSQWSGVDV	QSLLHSTGYNY	LGS	MQALEIPRLT
7s0b	GFTFSSYA	ISGSGGST	ARDLWGSGFFAFDV	QDISNY	DAS	QQDAGTPLT
7s11	GLSLTTNS	IWSNGGT	ARNFPYPGINFD	TGAVTTSNY	GTS	SLWYSGHLI
7s13	GLSLTNNI	IWSNGGT	ASRDYPGFAY	ELPKRY	EDS	LSTYSDDKLPI
7seg	GYTFTSYY	IEPMYGST	ARGSAYYYDFADY	NIGSKN	QDN	QVWDNYSVL
7sem	GFTFSSYS	ISASSSYS	ARARATGYSSITPYFDI	SSNIGAGYD	DNN	QSYDRSLSGV
7shu	GYNITSGYS	VTYDGST	AKGNNYFGHWHFAV	KSVDSDGDSY	AAS	QQSHEDPYT
7shz	GYSITSGYS	IKYSGET	ARGSHYFGHWHFAV	KPVDGEGDSY	AAS	QQSHEDPYT
7si0	GYSITSGYS	VTYDGST	ARGSHYFGHWHFAV	QSVDSDGDSY	AAS	QQSHEDPYT
7so9	ASGFTFNSYGMH	AFIRYDGGNKYY	YCANLKDSRYSGSYYDYWG	CQASQDIRFY	LLISDAS	ATYYCQQYDNLPF
7stz	VSGFSLSRYGVH	GMMWGGGNTDY	YCASSNYVLGYAMDYWG	CKSSQSLLNSSNQKNY	LLIYFTS	ADYFCQQHYRTPH
7vux	GFAFSSYD	ISGGGRYT	ASPYGGYFDV	QSISNF	YAS	QQSNSWPHT

Supplementary information 1. An example HADDOCK configuration file for docking

```
# ======================================================
# Example antibody-antigen HADDOCK docking workflow
# ======================================================

# run directory
run_dir = "antigen-AFL-Para-Epi-full-1000-200"
mode = "local"
ncores = 24
clean = true

#Input proteins
molecules =  [
    "ensemble_PDB_emref.pdb",
    "PDB_antigen_haddock-ready.pdb"
    ]
# ======================================================
# Parameters for each stage of the workflow
# ======================================================

[topoaa]

[rigidbody]
# CDR to surface ambig restraints
ambig_fname = "PDB_ambig_Para_Epi.tbl"
# Restraints to keep the antibody chains together
unambig_fname = "PDB_unambig_AF2.tbl"
sampling = 1000

[caprieval]
reference_fname = " PDB_target.pdb"

[seletop]
select = 200

[flexref]
tolerance = 20
# CDR to surface ambig restraints
ambig_fname = "PDB_ambig_Para_Epi.tbl"
# Restraints to keep the antibody chains together
unambig_fname = "PDB_unambig_AF2.tbl"

[emref]
tolerance = 5
# CDR to surface ambig restraints
ambig_fname = PDB_ambig_Para_Epi.tbl"
# Restraints to keep the antibody chains together
unambig_fname = "PDB_unambig_AF2.tbl"

[caprieval]
reference_fname = "PDB_target.pdb"

[clustfcc]
min_population=4

[caprieval]
reference_fname = "PDB_target.pdb"

# ======================================================
```

Note that in a real case, the reference structure won't be known and the "`reference_fname`" lines in the "`caprieval`" modules should be removed or commented out.
